# Sex-specific attenuation of constant light-induced memory impairment and *Clock* gene expression in brain in hepatic *Npas2* knockout mice

**DOI:** 10.1038/s41598-025-92938-1

**Published:** 2025-03-11

**Authors:** Ruby Chrisp, Mitchell Masterson, Rebecca Pope, Christopher J. Roberts, Hilary M. Collins, David J. G. Watson, Derek O’Neil, Kjersti M. Aagaard, Claire L. Gibson, David M. Heery, Paula M. Moran

**Affiliations:** 1 Gene Regulation and RNA Biology Laboratory, School of Pharmacy, BioDiscovery Institute, University Park, University of Nottingham, Nottingham, NG7 2RD UK; 2 School of Life Sciences, Queen’s Medical Centre, University of Nottingham, Nottingham, NG7 2UH UK; 3 Division of Maternal-Fetal Medicine, Departments of Obstetrics and Gynecology, Baylor College of Medicine and Texas Children’s Hospital, Houston, TX 77030 USA; 4 Department of Molecular and Human Genetics, Bioinformatics Research Laboratory, Baylor College of Medicine, Houston, TX 77030 USA; 5 School of Psychology, University Park, University of Nottingham, Nottingham, NG7 2RD UK

**Keywords:** Neuroscience, Psychology

## Abstract

NPAS2 (Neuronal PAS Domain Protein 2) is a component of the core circadian clock and the coordinated activity between central brain and peripheral liver clock proteins postulated to be instrumental for linking behaviour and metabolism. We investigated a conditional liver-specific knockout mouse model (*Npas*2-/- or cKO) to explore its function in activity, circadian rhythms and cognition (novel object recognition-NOR). Circadian rhythms showed no genotype differences. Constant-light reduced NOR in floxxed controls but remarkably not in *Npas*2-/- mice, particularly females. Consistent with entrainment of systemic and central circadian biology, *Npas*2-/- mice showed altered expression of circadian gene *Clock* in frontal cortex. Sex differences independent of genotype were found in expression of circadian genes *Clock*,* Bmal1* and *Reverb-b* in brain. Sex differences in *Clock* were absent in *Npas*2-/- mice. Females showed greater period length and phase response to constant light independently of genotype. The data suggest that a role for peripheral NPAS2 in constant light-induced memory impairment in females, and potential mediation by altered cortical circadian *Clock* gene expression, merit further investigation. These findings have implications for the interaction between peripheral and central circadian clocks, circadian sex differences and the deleterious effects of constant light on cognition.

**Supplementary Information:**

The online version contains supplementary material available at 10.1038/s41598-025-92938-1.

## Introduction

*Npas*2 is a circadian gene identified in the mammalian forebrain, where it functions as part of the molecular clock^1^. Circadian rhythms are highly conserved biological oscillations with a period of approximately 24 h. They have been identified in a multitude of tissues and are hierarchically controlled by the suprachiasmatic nucleus (SCN) within the anterior hypothalamus. An essential characteristic of these rhythms is their ability to be entrained by external stimuli, such as light and temperature, known as zeitgebers^2^. The SCN receives day/night cues from light through the retina, which entrains the central clock and results in the SCN orchestrating phase synchronisation of clocks in peripheral tissues. This allows for temporal coordination between tissues and rhythmic regulation of endogenous processes. Such processes include: the sleep/wake cycle, body temperature, and hormone secretion^3^. However, it is unknown whether the peripheral circadian machinery may paradoxically regulate central regulators, and what role this may play in higher cognitive function.

Circadian rhythms are generated by negative transcription-translation feedback loops (TTFLs) which involve post-translational modifications of transcriptional repressors^2^. The positive limb of the clock consists of three transcription factors: circadian locomotor output cycle kaput (CLOCK), neuronal PAS domain protein 2 (NPAS2) and brain and muscle ARNT-Like 1 (BMAL1)^4^. CLOCK and BMAL1, the principal components of the molecular clock, form heterodimers that act as transcriptional activators by binding E-box elements in core circadian genes *Period* (*Per1/Per2/Per3*) and *Cryptochrome* (*Cry1/Cry2*), as well as other clock-controlled genes. PER and CRY then heterodimerise and translocate back to the nucleus to repress their own transcription via inhibition of CLOCK: BMAL1 heterodimer activity^3^. It was subsequently shown that the transcription factor NPAS2 can also form complexes with BMAL1, similarly initiating transcription of clock-controlled genes in both central and peripheral tissues; NPAS2 is therefore a compensatory paralog of CLOCK^4^. Dysregulation of NPAS2 can lead to disruptions in circadian rhythms and may contribute to sleep abnormalities, cancer, cardiovascular and cerebrovascular diseases^5^.

In addition to (or possibly as a result of its role in circadian regulation) there is evidence that *Npas2* may also have other central nervous system behavioural functions in anxiety, cognition, and memory. Mice globally deficient in NPAS2 show deficits in long-term memory (LTM) during cued and contextual fear (CCF) tasks. N*pas*2 knockout mice display reduced freezing responses to both tone and conditioning context in comparison to control mice. This suggests that NPAS2 may be a requirement for conditioned fear responses and that NPAS2 loss impairs LTM in emotion-based tasks^6^. NPAS2 is highly enriched in dopamine D1 receptor (DRD1) containing neurons^7^. These neurons are of high density in the striatum, the nucleus accumbens and the substantia nigra. Importantly, DRD1-containing neurons have been implicated in a wide range of behaviours including the modulation of learning and memory^8–10^.

In addition to memory, NPAS2 has been suggested to have a role in anxiety and mood. *Npas2* null mutant mice display significantly reduced anxiety phenotypes exemplified by increased time spent in the open arms in elevated plus maze tests and significantly reduced latency to enter the light side of a light-dark box^7^. Furthermore, acute and chronic stress has been shown to increase striatal *Npas2* expression, suggesting that N*pas*2 is associated with stress responses in the striatum. Taken together, this evidence suggests loss of *Npas2* is anxiolytic and suggests a role for *Npas2* in the regulation of cognition and anxiety^7,10,11^.

Initially, it was theorised that the SCN was the only circadian pacemaker within the mammalian body, and that peripheral tissue timing was determined by the central clock. After demonstration of autonomous clocks in the liver, heart, and adrenal glands, a new hypothesis emerged, whereby peripheral, self-governing clocks may be present in all tissues^12–14^. The hepatic circadian clock is pivotal for carbohydrate, lipid and amino acid metabolic processes, oxidative pathways and detoxification^13,15,16^. The liver regulates blood glucose levels based upon a diurnal feeding/fasting schedule of dietary glucose intake. Loss of *Bmal1* in the liver, and the subsequent impacts on local circadian rhythmicity results in hypoglycaemia during the fasting phase, and loss of rhythmic glucose regulatory gene expression^17^. Yet, it is currently unknown how local loss of secondary circadian proteins in the liver, such as *Npas2* impacts global circadian rhythmicity.

In these experiments we investigated the loss of NPAS2 in the liver in object recognition memory and indices of circadian rhythmicity of locomotor activity. These experiments were carried out as a control condition to compare with a line of brain specific NPAS2 deletion mice intended to be subsequently derived from this line, with the hypothesis that deletion of liver NPAS2 would have no effect on recognition memory or general behaviour but may alter circadian rhythmicity of locomotor activity. We demonstrated that hepatic NPAS2 is not essential to maintain circadian rhythmicity of locomotor activity. Remarkably however, we identified behavioural changes in hepatic N*pas2 -/-* mice in novel object recognition task that we continued to investigate under varying lighting conditions, and by sex. To identify a possible mechanism for effects demonstrated we investigated circadian gene expression in the frontal cortex and found alterations in *Clock* as well as sex specific differences in *Clock Bmal1* and *Reverb-b.*

## Results

### *Npas2* cKO and lighting condition have effects on long-term recognition memory

Novel object recognition (NOR) tasks were performed to assess whether NPAS2 loss in the liver affected cognitive function.

**Fig. 1:**
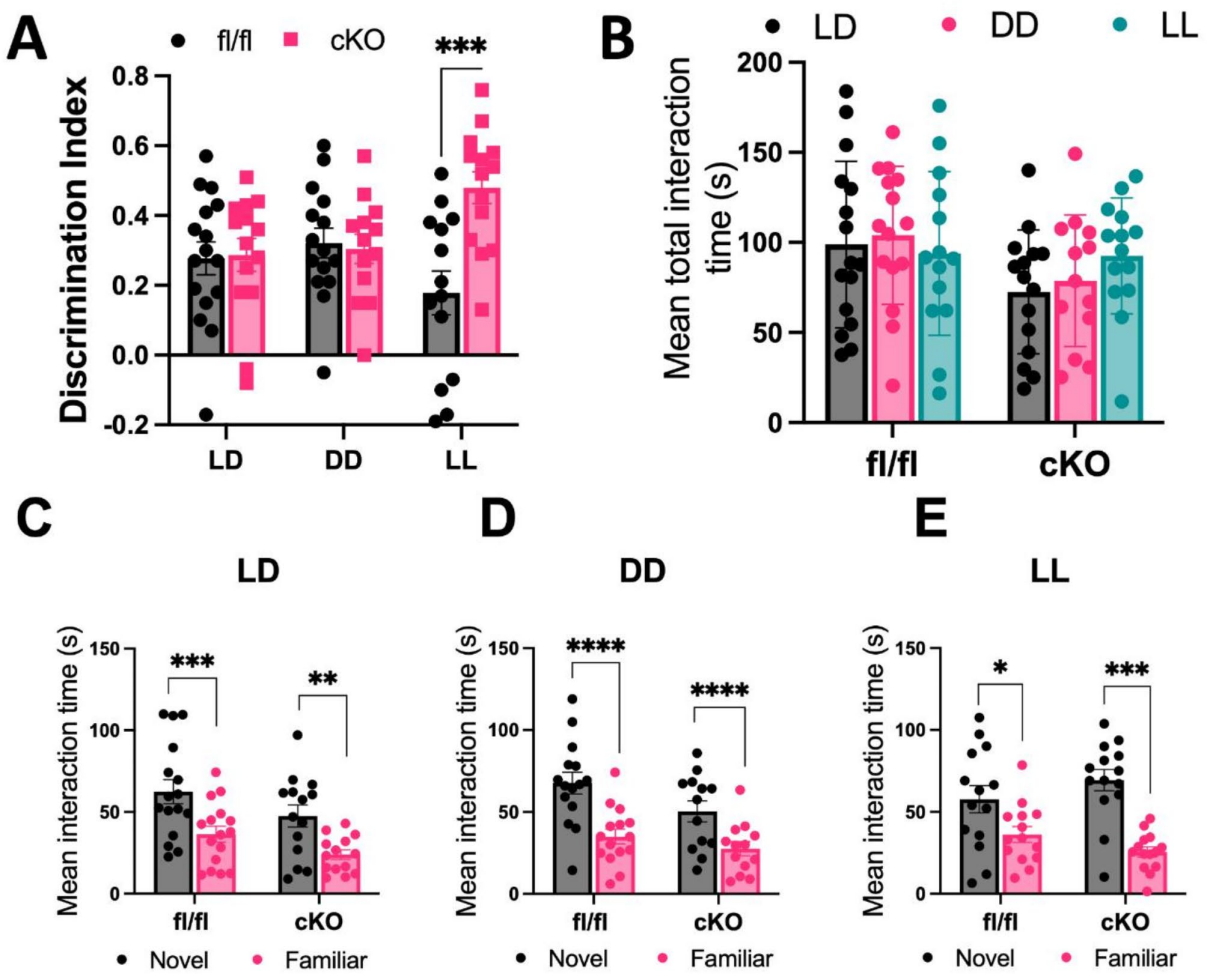
Object recognition memory differs in LL between *Npas2* cKO and fl/fl mice, but test interaction does not. (**A**) Discrimination indices (DI) of cKO (*n* = 14 all conditions) and fl/fl mice (LD *n* = 16, DD *n* = 15, LL *n* = 14). cKO mice had a significantly higher DI in LL compared to fl/fl (***p = 0.0001) and compared to cKO in LD (*p = 0.0181) and DD (*p = 0.0394). (**B**) Mean total interaction time of cKO (*n* = 14 all conditions) and fl/fl (LD *n* = 16, DD *n* = 15, LL *n* = 14). A significant effect of lighting condition was seen (*p = 0.0278), but no differences between genotypes were noted. (**C**) Mean interaction time with familiar and novel objects in cKO and fl/fl mice in: (**A**) LD conditions, cKO (*n* = 14) and fl/fl (*n* = 16) both preferentially explored the novel object (cKO: ***p = 0.0003, fl/fl: **p = 0.0034), (**B**) DD conditions, cKO (*n* = 14) and fl/fl (*n* = 15) mice both preferentially explored the novel object (cKO: ****p < 0.0001, fl/fl: ****p < 0.0001, ), (**C**) LL conditions, cKO (*n* = 14) and fl/fl (*n* = 14) mice both preferentially explored the novel object (cKO: *p = 0.0226, fl/fl: ***p = 0.0008).

Memory performance was measured by discrimination index (DI), which either showed a positive preference for novelty, or a negative preference for familiarity. Mixed-effects analysis revealed a significant effect of genotype (*F*(1,80 = 6.141, *p* = 0.0153) and a significant interaction between genotype and lighting condition (*F*(2,80) = 6.548, *p* = 0.0023) (Fig. 1A). DI’s revealed almost identical preferences for novelty between cKO and fl/fl controls in LD and DD, which were also comparable across both lighting conditions, however, cKO showed a significantly higher mean DI than fl/fl controls in LL, (Šídák’s post-hoc, *p* = 0.0001) (Fig. 1A) which is indicative of superior recognition memory. Intra-genotype differences revealed no significant difference in fl/fl memory performance across lighting conditions but did show that cKO had a significantly higher DI in LL than in DD (Tukey’s post-hoc, *p* = 0.0394) and LD (Tukey’s post-hoc, *p* = 0.0181) (Fig. 1A). Mean total interaction time, where interaction with both the novel and the familiar object were combined, revealed no significant differences in total test interactivity between genotypes, though a significant effect of lighting condition was seen (*F*(1,29) = 4.194, *p* = 0.0497) (Fig. 1B).

Both cKO and fl/fl mice displayed significantly more interaction with the novel object than the familiar object in all conditions (LD: Šídák’s post-hoc, fl/fl: *p* = 0.0034, cKO: *p* = 0.0003, DD: Šídák’s post-hoc, both: *p* < 0.0001, LL: Šídák’s post-hoc, fl/fl: *p* = 0.0008, cKO: *p* = 0.0226) (Fig. 1C, D and E). This increased interaction with the novel object was significantly above chance in both genotypes, under all lighting conditions (LD: fl/fl *t*(15) = 5.893, *p* < 0.0001, cKO *t*(13) = 6.103, *p* < 0.0001), (DD: fl/fl *t*(14) = 7.554, *p* < 0.0001, cKO *t*(12) = 7.342, *p* < 0.0001), (LL: fl/fl *t*(13) = 2.837, *p* = 0.0140, cKO *t*(13) = 10.50, *p* < 0.0001). No spatial bias or object preferences were noted in sample trials (Figure S2). Taken together these data suggest a light condition specific improvement of long-term recognition memory performance in NPAS2 cKO mice, that is unlikely to be explained by increased total object interaction time.

### Locomotor activity and circadian rhythmicity was not altered in hepatic ***Npas2*** -/- mice

**Fig. 2:**
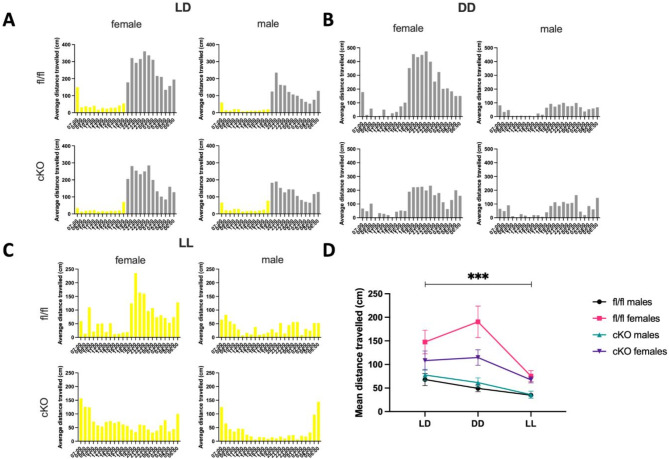
Average hourly activity patterns do not differ between cKO and fl/fl mice, and both reduce their activity in LL. (**A**) Average hourly activity of fl/fl (*n* = 16) and cKO (*n* = 16) taken over 5 days in LD cycling. (**B**) Average hourly activity of fl/fl and cKO taken from one day at the end of DD conditions. (**C**) Average hourly activity of fl/fl and cKO taken from one day at the end of LL conditions. (**D**) Mean hourly distance travelled. LD represents average hourly activity over a 5-day period. DD and LL represents the final 48-hours of each condition. Both genotypes showed a reduction in activity in LL compared to LD (fl/fl *p = 0.0406, cKO *p = 0.0294) or DD (fl/fl *p = 0.0115, cKO *p = 0.0326).

To assess whether differences in recognition memory performance could be confounded by altered general locomotor activity, distance travelled and activity patterns were measured between the two genotypes. Locomotor activity recordings of distance travelled were taken in 10-minute bins during all three lighting treatments. No significant differences were noted in the average hourly activity patterns of each genotype in LD, DD or LL conditions. In LD, for both fl/fl controls and cKO, onset of activity begun almost as soon as the lights were turned off at ZT12 and activity ended immediately after the lights were turned back on at ZT0. Locomotor activity in both the light phase and the dark phase was comparable between the same sex of opposing genotypes. Though, both female groups demonstrated increased locomotor activity compared to males throughout (Fig. 2A). Following the onset of DD, all groups became generally more active across a 24-hour period, with activity dispersing into the subjective day. Here, fl/fl females displayed increased distance travelled in comparison to cKO females (Tukey’s post-hoc, *p* = 0.0470) (Fig. 2B). After the induction of LL, fl/fl females displayed reduced distance travelled in comparison to DD (Tukey’s post-hoc, *p* < 0.001), yet this effect was not seen in cKO females or males of either genotype (Fig. 2C and D). Three-way ANOVA analysis revealed a significant effect of lighting condition on locomotor activity (*F*(2, 138) = 7.875, *p* = 0.0006) (Fig. 2C and D). Additionally, there was a significant genotype X sex X lighting condition interaction(*F*(2, 138) = 3.373, *p* = 0.0371) and a significant effect of sex alone (*F*(1, 138) = 101.5, *p* < 0.0001). Genotype did not have a significant effect on locomotor activity. Actograms generated using this distance travelled data can be found in the supplementary data (Figure S4, S5, S6, S7).

### No significant differences were seen in circadian period, phase or amplitude in hepatic ***Npas2*** -/- mice

**Fig. 3:**
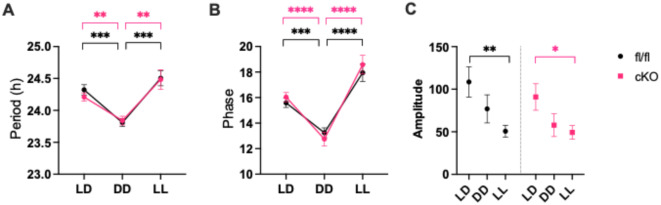
No significant differences in circadian parameters between cKO and fl/fl controls in any lighting condition. (**A**) No significant difference was seen in period length between cKO (LD *n* = 16, DD and LL *n* = 15) and fl/fl controls (*n* = 16 in all) in any lighting conditions. Period significantly decreased in DD in both genotypes (fl/fl: ***p = 0.0003, cKO: **p = 0.0107), and significantly increased in LL in both genotypes (fl/fl: ***p = 0.0002, cKO: **p = 0.0049). (**B**) No significant differences in phase were seen between genotypes in any lighting condition. Phase was significantly lower in DD in both genotypes (fl/fl: ***p = 0.0002, cKO: ****p < 0.0001) and was significantly higher in LL in both genotypes (both: ****p < 0.0001). (**C**) No significant differences in amplitude were seen between genotypes in any lighting condition. Amplitude was significantly reduced in LL compared to LD in both fl/fl (**p = 0.0031) and cKO mice (*p = 0.0475).

Locomotor activity recordings were then examined using FFT-NLLS cosine analysis to yield period, phase and amplitude estimates. This was to establish whether poorer recognition memory performance in cKO mice during LL conditions could be explained by altered circadian rhythmicity. Mixed-effects analysis revealed lighting condition significantly impacted circadian period (*F*(2,87) = 24.02, *p* < 0.0001), but genotype did not (*F*(1,87) = 0.1936, *p* = 0.6610). DD shortened circadian period in both genotypes (Šídák’s post-hoc, fl/fl: *p* = 0.0003, cKO: *p* = 0.0107) and LL elongated circadian period in both genotypes (Šídák’s post-hoc, fl/fl: *p* = 0.0002, cKO: *p* = 0.0049) (Fig. 3A).

FFT-NLLS cosine analysis also revealed that circadian phase was significantly influenced by lighting condition (mixed effects analysis, *F*(1.575,44.89) = 61.82, *p* < 0.0001)), but not by genotype (*F*(1,30) = 0.1896, *p* = 0.6664). Both genotypes demonstrated a significant phase advance in DD (Šídák’s post-hoc, fl/fl: *p* = 0.0002, cKO: *p* < 0.0001), and a significant phase delay in LL (Šídák’s post-hoc, both *p* < 0.0001) (Fig. 3b). Similarly, rhythm amplitude was significantly impacted by lighting condition (mixed effects analysis, *F*(2,60) = 9.047, *p* = 0.0004), but not by genotype (*F*(1,30) = 0.8636, *p* = 0.3604). Both fl/fl and cKO mice demonstrated reduced amplitude in LL compared to LD (Šídák’s post-hoc, fl/fl: *p* = 0.0031, cKO: *p* = 0.0475) (Fig. 3c). Thus, all core circadian parameters remain unchanged despite the loss of NPAS2 in the liver, suggesting hepatic NPAS2 is not required for global rhythmicity, as hypothesized.

### Anxiety phenotypes in hepatic ***Npas2*** -/- mice

Next, to assess if recognition memory differences between fl/fl and cKO mice may be due to an anxiety phenotype following hepatic NPAS2 loss as has been demonstrated previously in a global Npas2-/- model, both groups were subjected to open field arena and light-dark box anxiety trials. No anxiety phenotype was seen in any parameter in either anxiety paradigm (Figures S5, S6).

### Genotype variation in recognition memory and sexual dimorphism in fl/fl control mice

**Fig. 4:**
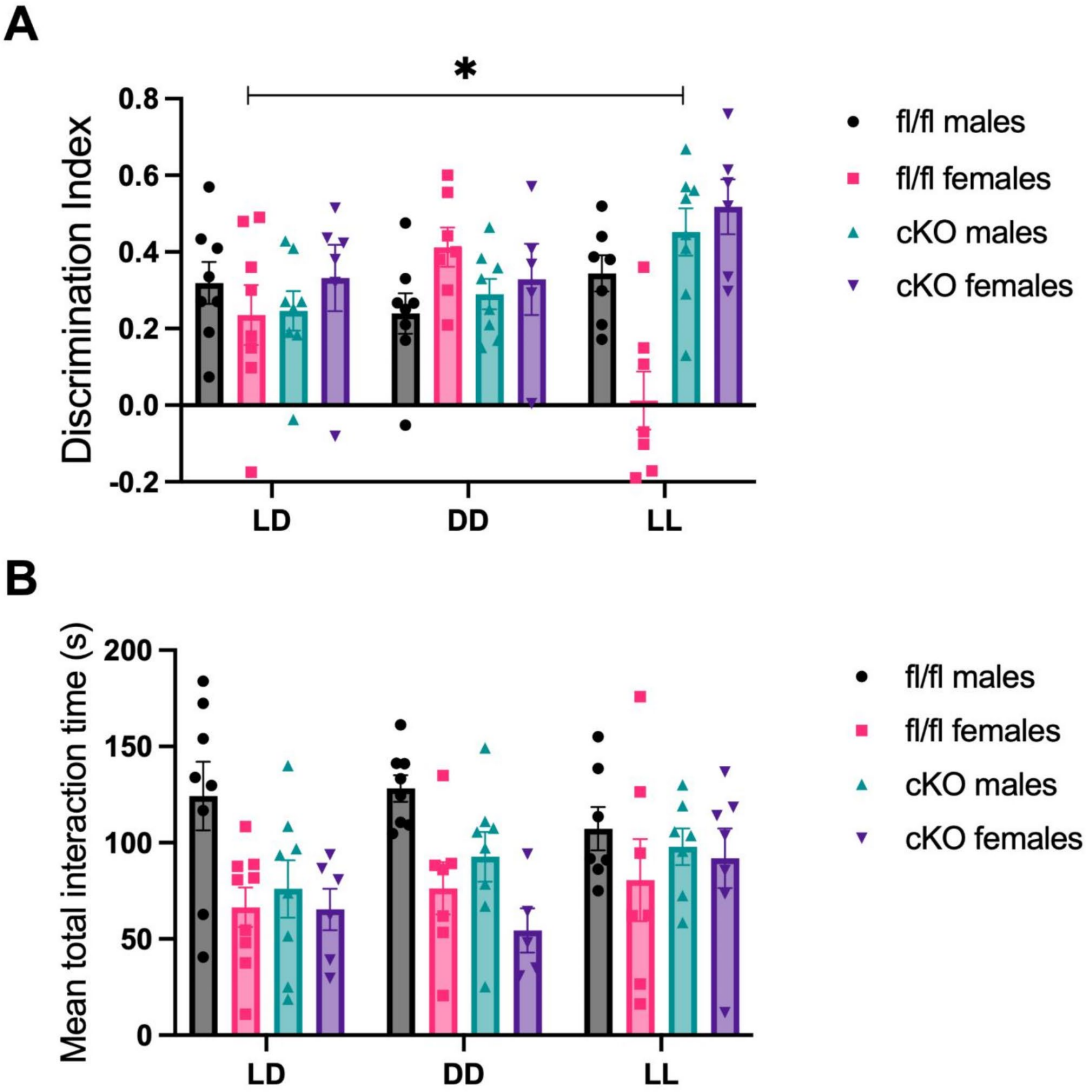
Fl/fl females have reduced recognition memory in LL compared to cKO females. (**A**) There was a significant sex X genotype X lighting condition interaction on DI (*p* = 0.0178). Fl/fl females (LD *n* = 8, DD and LL *n* = 7) showed a significantly worse DI in LL than cKO females (LD, DD *n* = 6, LL *n* = 7) (*p = 0.0153). Fl/fl males (LD and DD *n* = 8, LL *n* = 7) and cKO males (LD, DD *n* = 8, LL *n* = 7) showed no significant differences in DI in any lighting condition. (**B**) Mean total interaction time in all lighting conditions. A significant effect of sex was seen (**p = 0.0025), but no individual effects of sex are noted.

While previous literature is variable, some studies have shown object recognition memory can vary by sex^18–20^. Since variation in recognition memory performance in LL between genotypes was not explained by altered anxiety phenotypes, or activity levels, an exploratory analysis of sex on DI was performed. A significant interaction sex X genotype X lighting condition interaction was seen on DI (mixed effects analysis, *F*(2,74) = 4.256, *p* = 0.0178). There were also significant lighting condition X sex (*F*(2,74) = 3.442, *p* = 0.0372), lighting X genotype (*F*(2,74) = 7.804, *p* = 0.0008) interactions and an effect of genotype (*F*(1,74) = 7.463, *p* = 0.0079). Notably, post-hoc analysis revealed a significant difference in LL DI between cKO females and fl/fl females, where cKO females showed significantly better recognition memory (Tukey’s post-hoc, *p* = 0.0153) (Fig. 4A).

In addition, total interaction time was also assessed for an impact of sex. There was no significant interaction between sex, genotype and lighting condition (*F*(2,47) = 0.4964, *p* = 0.4399). However, an effect of sex was seen (*F*(1,28) = 11.03, *p* = 0.0025). Males appeared to interact with the novel object test more than females. Despite this, no individual effects in any lighting condition were seen between sexes, and no effect of genotype was noted (Fig. 4B). Furthermore, no differences in total test interaction were observed between cKO females and fl/fl females in any lighting condition. Thus, any variance in memory performance between cKO and fl/fl females is unlikely to be attributable to altered engagement with the task.

### Sexual dimorphism in circadian period in conditions of constant light

**Fig. 5:**
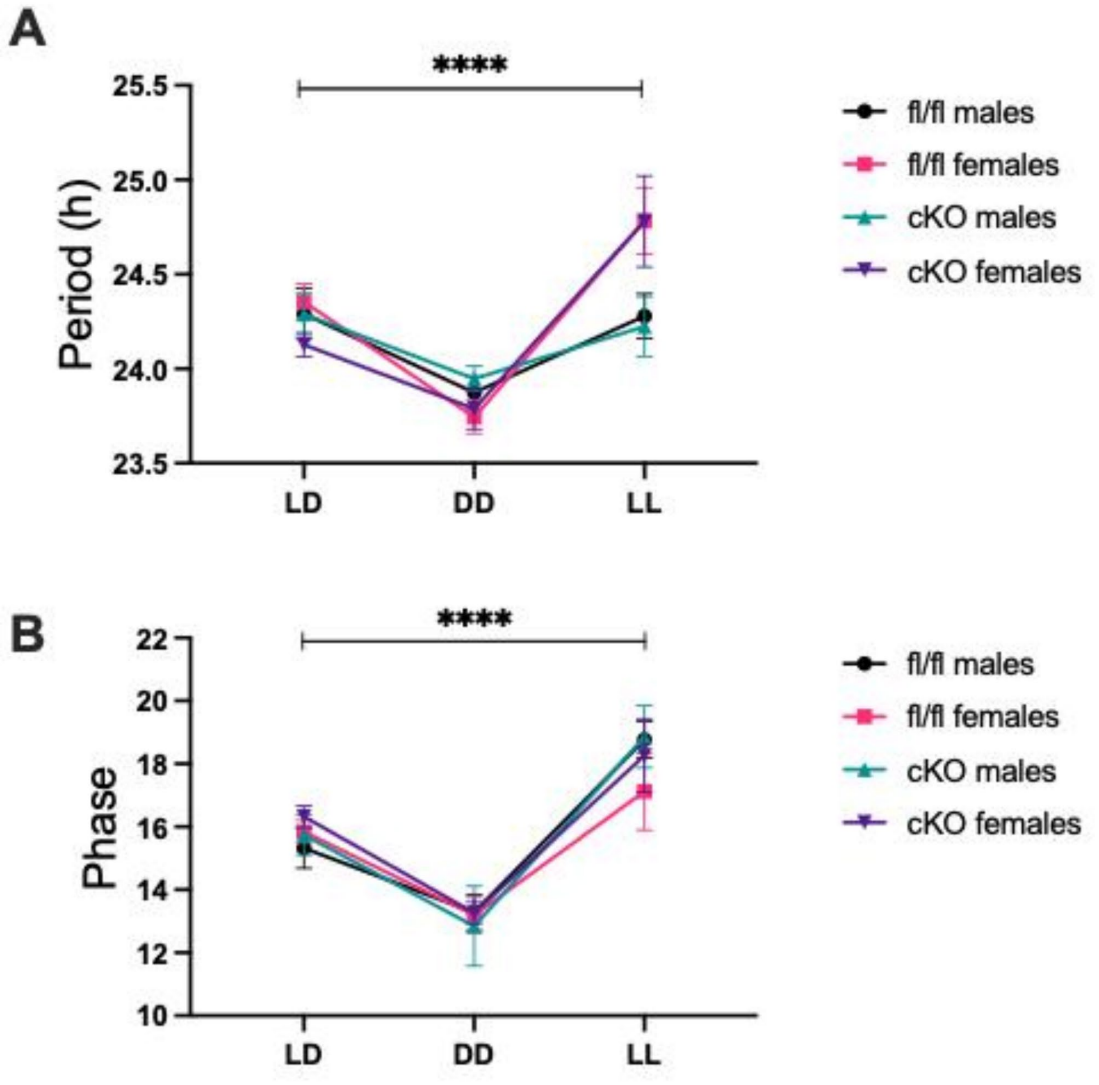
Sexual dimorphism is present in circadian period across genotypes. (**A**) A significant effect of lighting condition was seen on period length (****p < 0.0001) across fl/fl males (*n* = 8), fl/fl females (*n* = 8), cKO males (LD, LL *n* = 8, DD *n* = 7) and cKO females (LD, DD *n* = 8, LL *n* = 7). A significant interaction of lighting condition and sex was also seen on period length (***p = 0.006). (**B**) A significant effect of lighting condition was seen on phase (****p < 0.0001) across fl/fl male and females, and cKO male and females.

Circadian parameters were also then analysed to assess whether recognition memory changes observed may be due to sex specific changes in circadian period and phase. No interaction effect of sex X genotype X lighting condition was seen. However, there was and a significant effect of lighting condition on circadian period (*F*(1.492, 61.16) = 28.44, *p* < 0.0001) and a significant interaction between lighting condition and sex (mixed effects analysis, *F*(2,82) = 8.106, *p* = 0.0006). Most notably, period was increased in females compared to males irrespective of genotype (Fig. 5A).

An effect of lighting condition was found on phase (mixed effects analysis, *F*(1.588, 42.88) = 54.15, *p* < 0.0001), but there was no effect of sex or interactions with sex (Fig. 5B).

### Central circadian gene expression in hepatic ***Npas2*** -/- mice

**Fig. 6:**
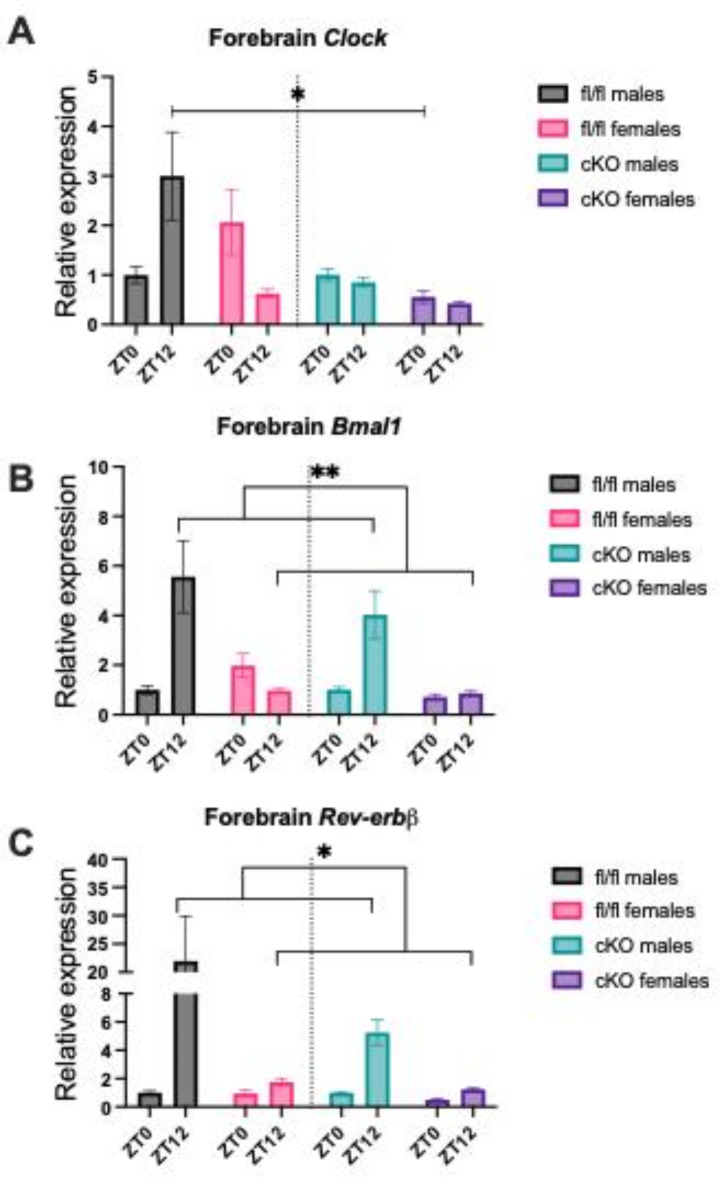
Sex, and hepatic *Npas2* expression, can influence central circadian gene expression in the forebrain. (**A**) A significant effect of genotype (**p = 0.0068), a significant interaction between time point and sex (*p = 0.0152), and a significant interaction between time point X genotype X sex (*p = 0.0145) were seen on *Clock* gene expression in the forebrain. (**B**) A significant effect of time point (**p = 0.0059), a significant effect of sex (**p = 0.0040), and a significant interaction between time point and sex (***p = 0.0008) were seen on *Bmal1* gene expression in the forebrain. (**C**) A significant effect of time point (*p = 0.0105), a significant effect of sex (**p = 0.0187), and a significant interaction between time point and sex (*p = 0.0242) were seen on *Rev-erbβ* gene expression in the forebrain. *n* = 10 per genotype (*n* = 4 female, *n* = 6 male for cKO and fl/fl).

Next in order to examine potential mechanisms of NOR effects identified transcript levels of core circadian genes in the frontal cortex were analysed between genotypes and sex groups using RT-qPCR. This was measured at ZT0 and ZT12, to additionally assess if cyclical gene expression was maintained in hepatic *Npas2* cKO mice. Previous findings in global *Npas2* KO mice have noted dampened oscillatory gene expression in some circadian targets^1,2^. A significant effect of time point X genotype X sex was seen on frontal cortical *Clock* expression (Three-way ANOVA, *F*(1,40) = 6.535, *p* = 0.0145), as well as a significant interaction between time point and sex (*F*(1,40) = 6.434, *p* = 0.0152). *Clock* expression was seen to be antiphase between fl/fl females and fl/fl males. Fl/fl males had a significantly higher central *Clock* gene expression at ZT12 than fl/fl females (Tukey’s post-hoc, *p* = 0.008). In addition, reduced expression was seen in both cKO sexes, and a significant effect of genotype was noted (*F*(1,40) = 8.161, *p* = 0.0068) (Fig. 6A).

Frontal cortical expression of *Bmal1* at ZT0 and ZT12 revealed significant effects of sex (Three-way ANOVA, *F*(1,39) = 9.345, *p* = 0.0040) and time point (*F*(1,39) = 8.493, *p* = 0.0059), as well as a significant interaction between sex and time point (*F*(1,39) = 13.37, *p* = 0.0008). As with frontal cortical *Clock* expression, *Bmal1* expression appeared to be antiphase between fl/fl males and fl/fl females. At ZT12, central *Bmal1* expression was significantly higher in fl/fl males compared to fl/fl females (Tukey’s post-hoc, *p* = 0.0027). cKO males displayed a similar pattern of *Bmal1* expression as fl/fl males, peaking at ZT12, but fl/fl females appeared to show no changes in expression over the two time points (Fig. 6B).

Finally, frontal cortical expression of *Rev-erbβ* at ZT0 and ZT12 was assessed. All genotype and sex groups displayed in phase *Rev-erbβ* expression, with peaks at ZT12. A significant effect of time point was noted (Three-way ANOVA, *F*(1,44) = 6.956, *p* = 0.0115), as well as a significant effect of sex (*F*(1,44) = 5.957, *p* = 0.0187). Importantly, a significant interaction between sex and time point was seen (*F*(1,44) = 5.453, *p* = 0.0242). Both cKO and fl/fl females display reduced ZT12 *Rev-erbβ* expression compared to male counterparts (Tukey’s post-hoc, fl/fl: *p* = 0.0037) (Fig. 6C). This suggests males have stronger *Rev-erbβ* oscillatory gene expression. No effect of genotype was noted on *Rev-erbβ* expression, though fl/fl males demonstrated higher levels at ZT12 than cKO males (Tukey’s post-hoc, *p* = 0.0134).

## Discussion

These data demonstrate (1) novel object memory was impaired under constant light conditions in female controls, but this impairment was absent in hepatic *Npas*2 knockout mice (2) Hepatic *Npas2* mice showed concomitant altered expression of the circadian genes in brain. (3) Genotype independent sex differences were found in period length changes in response to different lighting conditions and day/night expression of circadian genes *Clock*,* Bmal1* and *Reverb-b.* (4) No changes in circadian period length or phase in activity were noted in N*pas2 -/-* mice compared to controls.

### Novel object recognition in constant light (LL)

Novel object recognition memory was found to be present in both genotypes in LD and DD, with no significant effects of genotype or lighting condition. In constant light conditions (LL) *Npas2 -/-* mice showed a significantly better performance compared to fl/fl controls. This is a particularly surprising finding inferring loss of NPAS2 in the liver can potentially influence recognition memory. It has been found previously that constant light negatively influences learning and memory in nocturnal rodents, with most noted deficits seen in spatial memory^21–24^ but impairment has also been shown in object discrimination paradigms^25^. As LL significantly affected fl/fl period and phase, it is reasonable to assume the lighting condition was effective in our experimental conditions. *Npas2 -/-* mice were less affected by LL-induced memory deficits and demonstrated a significant improvement in recognition memory compared to other lighting conditions. This contrasts with prior studies of cognition in global N*pas2 -/-* mice which have been shown to have specific impairment in contextual fear-based long-term memory suggesting specific types of memory and their environmental moderation may differentially involve NPAS2. It should be noted that since alterations in anxiety have also been found in global *Npas2 -/-* mice in those studies, it may have been difficult to isolate anxiety as a mediating variable^6^.

Interestingly, there were no improvements or deteriorations of recognition memory under DD conditions compared to LD, which incidentally suggest that NOR in these conditions is not a visuospatial task as is commonly assumed. This supports previous suggestions that NOR asks can be successfully performed using non-visual methods of novelty discrimination^25^. This contrasts with literature showing constant darkness impairs spatial memory in both Wistar rats and C57BL/6J mice but visuo-spatial versus non visuo-spatial strategies such as whisker proprioception may help to explain this difference. One other difference from prior studies apart from using spatial memory tasks is that the DD period they were exposed to was longer than four weeks and therefore memory impairments may require longer DD exposure^22,24^. Furthermore, the majority of these studies tested in light conditions following DD treatment, rather than maintaining the DD conditions for the test, as we did. This sudden change to bright light could potentially induce anxiety and/or memory impairment.

The effect on recognition memory we identified was seen predominantly in female mice. Previous findings are inconclusive on whether NOR tests show male, or female, or no superiority in mice^19,22,26^. In the present study, fl/fl and cKO mice demonstrate no sex differences in cycling conditions or in DD, which suggests no sex differences in recognition memory are present in 12 h day/night or free-running lighting. In constant light male fl/fl mice performed better than females. This sex difference was not seen in N*pas2 -/-* mice. To our knowledge, this has not been reported previously. The present findings are consistent with conclusions from previous studies identifying sex differences in light sensitivity and circadian cycles in humans^27–29^. This suggests that potential cognitive impairment in constant light conditions may be sex-dependent, and further, that hepatic NPAS2 deletion attenuates this impairment. Further experiments are required to test this hypothesis and should control for oestrus cycle. While there are reports that show no variation across the cycle on novel object recognition in C57BL/6 mice^30^ equally some that do suggest differences^31^.

These findings may have implications for understanding the effects of constant light on human health which is a burgeoning health issue due to shift work and light pollution in cities^32,33^. We reversed the order of the lighting conditions between cohorts to rule out any potential artefacts of order of light conditions as contributing to the results found. Both cohorts independently displayed significant differences in recognition memory in constant light and upheld the improved performance in cKO mice (supplementary Figure S10). Previous studies have shown exploration may confound interpretation of cognitive performance^19,26^. We noted no significant differences between genotypes in exploration time across lighting conditions. There was a significant overall sex difference in exploration. Males of both genotypes showed higher exploration in the NOR test which we hypothesised may be indicative of a higher-level anxiety in females, though female mice have generally been found to be more active than males^19,26,34,35^. We found no sex differences in exploration in constant light, suggesting exploration time is unlikely to have had an influence on altered novel object recognition performance in LL conditions identified. As global *Npas2 -/-* mice have been shown in previous studies to have reduced anxiety in open field tests and elevated plus maze trials, and specific NPAS2 knockdown in the ventral striatum using viral vector approaches also produces anxiolytic effects, we assessed if hepatic *Npas2* loss affects anxiety, potentially mediating recognition memory changes^6,7,36^. No difference between *Npas2 -/-* and fl/fl control mice were seen in either LD box or open field anxiety tasks, suggesting that anxiety differences do not explain differences in cognitive performance in NOR.

### Circadian rhythms in locomotor activity

*Npas2 -/-* and fl/fl hourly locomotor activity depicted similar patterns of behaviour over all lighting conditions. There were no significant genotype differences in circadian period in any lighting condition. Both genotypes showed shortened periods in constant darkness, and elongated periods in constant light, as demonstrated previously in mice^36–38^. Mice exposed to DD typically display a phase advance, where their peak of activity moves to earlier in the subjective day^36,39,40^. Both cKO and fl/fl mice displayed a similar phase advance in DD. LL conditions also successfully provoked a significant phase delay that was equivalent between genotypes, which is characteristic of LL treatment^37,38^. This suggests that post-natal hepatic NPAS2 deletion does not have a significant effect on circadian parameters, either in entrainment or free-running conditions, confirming that peripheral rhythm perturbation does not significantly alter global rhythmicity^2^. However, there was a significant interaction between sex and lighting condition on period length. Specifically, a disparity in LL period can be seen between males and females of both genotypes. If fl/fl females were more significantly disrupted by the LL condition, this may have resulted in diminished recognition memory, since effective LL treatment has previously been noted to impair memory^21–24^. Though as the same effect was noted in cKO mice, this is unlikely to explain the genotype disparities observed.

### Circadian gene expression in brain

In order to identify a potential mechanism for sex-specific recognition memory effects following hepatic *Npas2* loss we examined day/night expression of circadian genes in brain. There were significant effects of sex and/or hepatic *Npas2* loss on *Clock*, *Bmal1* and *Rev-erbβ* expression in the frontal cortex. Antiphase patterns in fl/fl mice were noted in frontal cortical *Clock* gene expression, where opposing cycling meant fl/fl females had peak expression at ZT0, whilst fl/fl males had peak expression at ZT12, and neither cKO sex demonstrated cyclical expression. Previously, Dudley et al.,^36^ found that global *Npas2* loss demonstrated no significant alterations in *Clock* gene expression in the forebrain, however, this mouse cohort was all male. For *Bmal1* expression in the frontal cortex cKO females showed no cycling of *Bmal1* at ZT0 and ZT12 time points, unlike both males and fl/fl females. Previously, loss of *Bmal1* in the forebrain has been shown to result in NOR memory deficits. In contrast, *Bmal1* knockdown in microglia resulted in significant improvements in long-term recognition memory in the NOR paradigm^41,42^. Since cKO females demonstrate loss of cyclical *Bmal1* expression between ZT0 and ZT12, and significantly improved memory performance from fl/fl females in LL, there may be a mechanism by which aberrant *Bmal1* expression improves cognitive output. Additionally, this presents fl/fl females as having irregular forebrain circadian gene expression compared to all other groups tested, which may mechanistically help to explain their cognitive deficits. Loss of this anti-phase male versus female *Clock* and *Bmal1* expression may be why hepatic *Npas2 -/-* mice do not show sex differences in cognition under conditions of constant light. This hypothesis would need to be tested empirically. Furthermore, we demonstrate sexual dimorphism in central circadian gene expression. This corroborates sex differences in previous studies in both animals and human post-mortem brain^43–45^. It would be of interest in future experiments to investigate mRNA expression using increased time resolution points over 24 h.

Other potential mechanistic causes of sexual dimorphism in cognition and circadian gene expression worth exploration include lipid metabolism and the microbiome. Recently, abolishment of gut microbiota has been shown to eliminate sex specific liver metabolism and rhythmicity as a result of altered gonadal hormone secretion^46^. Similarly, sexual dimorphism in mouse liver metabolism and gonadal hormone release is thought to be reliant on circadian genes *Cry1*/*Cry2*^47^. Together, this proposes interconnected roles for the clock in sexual dimorphism of liver function, which may merit further study in relation to our data. This could help elucidate if a brain-liver axis which is capable of generating sexually dimorphic cognitive responses, has potential mechanistic reliance on the gut microbiome.

### Genotype-independent sex differences in circadian rhythms

Sexual dimorphism in free-running period is seen to be very species specific, with C57BL/6J mice showing no sex differences in free-running period^45,48–50^. Our findings agree with these studies, as we saw no sex differences in period or phase in entrainment. Our study did however identify significant sex differences in circadian phase and circadian period in constant light conditions. This raises the suggestion that adaptation to constant light conditions may differ between the sexes. Sex differences in adaptation to bright light has been identified previously in human studies in the context of understanding sex differences in adaptations to shift work^29^. Our findings suggest the hypothesis that frontal cortical *Clock* potentially via a mechanism that involves hepatic NPAS2 may play a role in such adaptations and merits further investigation. We cannot determine from these experiments how generalisable these findings are to other mammals or even other mouse species thus future experiments will be required to test the hypothesis raised. The behaviour of the Fl/Fl control mice here does show broad comparability in locomotor activity response to lighting conditions, anxiety and cognitive behaviour to wild-type C57BL6/j both in our hands and in previous studies (see supplementary).

### Summary and implications

In summary we have shown using a mouse model that hepatic NPAS2 may play a role in cognitive impairment consequent to constant light in females. One potential mechanism to be explored is through modulation of frontal cortical *Clock* gene expression. Manipulations that disrupt the circadian system such as SCN lesions or jet-lag have been shown to induce impairment in cognition [see 65 for review]. A number of neuropsychiatric disorders, including Alzheimer’s disease, depression, PTSD and ageing are associated with both cognitive impairment and circadian abnormalities, suggesting that these two biological processes are linked^51^. While a precise mechanism cannot be determined here the present data support this suggestion. A “gatekeeping function” of peripheral clocks has very recently been demonstrated where the epidermal clock been shown to gate brain clock signals to ensure epidermal health^52^. Additionally, the central clock drives the oscillations of the peripheral muscle clock which integrates these signals from the central clock to ensure tissue coherence^53^. These gatekeeping functions of peripheral over central clock suggests the idea that there is a decentralized network of clocks rather than a hierarchical, brain-centric network with the central clock alone controlling peripheral tissue clocks^53^. Here we show that it is possible for a peripheral circadian regulator to feedback to “correct” cognitive impairment induced by abnormal light conditions in a sex specific manner. This raises the possibility of a gatekeeping function between liver and brain clocks to regulate cognitive response to abnormal light conditions light in females. We suggest that peripheral hepatic NPAS2 and central cortical Clock merit further investigation as potential mechanisms.

## Materials and methods

### *Npas2* hepatic cKO mice

A breeding colony was set up using mice imported from the Aagaard group at the Baylor College of Medicine, TX (23). Transgenic line generation (on a C57BL6/j background) was described in O’Neil et al., 2017^54^. Mice used throughout these studies are *Npas2* ^fl/fl^/*Alb-Cre* ( referred to as *“*N*pas2 -/-” or “cKO”*), *and Npas2* ^*fl/fl*^ (referred to as *“fl/fl”*). Littermates of both sexes ( 4–6 months) were used throughout the experiments reported herein.

### In vivo husbandry and general care

Experimental and holding rooms were kept at 22^o^C ± 2^o^C with a humidity of 50–55%. All animals had *ad libitum* food and water access. All experiments were carried out in compliance with the U.K. Home Office regulations on animal experimentation, with protocols approved by University of Nottingham Animal welfare Ethics Board with appropriate personal and project license authority under the Animals (Scientific Procedures) Act, UK 1986. UK Home Office Project License No: P16F842EF. Authors confirm reporting complies with ARRIVE guidelines.

### Experimental set up and circadian recording

Locomotor activity recording was measured inside light-and sound-attenuated ventilated chambers (80 cm x 61 cm x 71 cm), which house four identical cages (16 cm x 19 cm x 26 cm). Each chamber was equipped with a fluorescent white bulb (150 lx), an infrared (IR) light and roof-mounted recording camera (ENV-018, MED Associates Inc, St Albans, VT, USA). The camera acts in conjunction with the Video Tracking Interface Software (Version 1, MED Associates Inc, VT, USA). The footage was converted into distance travelled (cm) by the Activity Monitor Software (Version 5, MED Associates Inc, VT, USA). Data was stored in 10-minute bins and analysed using BioDare2^55^. Distance travelled data was used to generate double plotted actograms using ActogramJ^56^. A radio played at 79 dB to mask any external sound cues from the building^57^. Experiments started with five days 12 h:12 h light/dark (LD) cycling entrainment, followed by 12 days of constant darkness (DD), and then 12 days of constant light (LL) at 150 lx.

### Novel object recognition (NOR) test protocol

The NOR protocol was adapted from Gresack et al., 2007^17^ and Zhao et al., 2012^58^. Tests were performed at 10am over three consecutive days at the end of the lighting condition treatment (days 5–7 of LD and days 10–12 of DD and LL). LD and LL tests were performed in light conditions of 150 lx, DD tests were performed in complete darkness. Day one: habituation – mice were removed from their home cages and placed inside arenas (16 cm x 19 cm x 26 cm) for five minutes to habituate to the new cage. Day two: sample trial – mice were re-habituated to the arena for one minute, then two identical objects were placed in the top left and bottom right corners of the arena for five minutes. A threshold of > 15 s interaction in all sample trials was set. Day three: test trial – mice were re-habituated to the arena for one minute, then one familiar object from day two and one novel object was placed in the top left and bottom right corners of the arena. All objects were washed in 10% cider vinegar solution to remove odour cues. Interaction with the objects was recorded for 10 min and manually scored. This was performed by two independent, blinded assessors. Inter-rater reliability was on average > 90% (Pearson’s correlation coefficient) and Cronbach’s alpha was > 0.94. A discrimination index (DI) was calculated by the following equation: DI = (tN-tF)/(tN + tF), where tN is time spent with the novel object, and tF is time spent with the familiar object. DIs give a value between negative one and positive one. Zero indicates no statistically significant preference. Negative values show a preference for the familiar object, and positive values show a preference for the novel object.

### Light-dark box protocol

The light-dark box protocol was adapted from Costall et al., 1989^55^, Heredia et al., 2014^56^, Kulesskaya and Voikar, 2014^57^, and Miller et al., 2011^59^. A clear, acrylic arena (16 cm x 19 cm x 26 cm) was split into two, equal sections. One section was brightly lit (150 lx) and the other section was completely dark (< 10 lx) and covered. A small opening between the two chambers allowed the mouse to transition. Mice were placed in the light chamber, and activity was recorded for five minutes. Results were analysed using manual timing by two independent, blinded assessments. Inter-rater reliability was on average > 99% (Pearson’s correlation coefficient) and Cronbach’s alpha was > 0.99.

### Open field arena protocol

A clear, acrylic arena (16 cm x 19 cm x 26 cm) was split into central and peripheral zones. Time spent in each zone and total distance travelled was recorded over a five-minute period. Time spent in each zone was measured using manual timing by two independent, blinded assessors. Total distance travelled was measured using Ethovision (Ethovision XT). Inter-rater reliability was on average > 80% (Pearson’s correlation coefficient) and Cronbach’s alpha was > 0.93.

### RNA isolation and quantitative PCR (qPCR)

Mice were culled at ZT0 and ZT12 time points during 12 h:12 h light/dark cycling by CO_2_ inhalation. ~80 mg of liver or frontal lobe were placed in 1 ml of cold Trizol reagent (Sigma), minced and sonicated for two x 15 s to disrupt the tissue. RNA isolation was then performed according to the manufacturer’s protocol using a Trizol/chloroform method (Sigma). cDNA synthesis was performed using the QuantiTect Reverse Transcription kit (QIAGEN Ltd), as per the manufacturer’s protocol. RTqPCR was performed using Brilliant II SYBR^®^ Green QPCR Master Mix from Agilent Technologies, as per the manufacturer’s protocol (Agilent Technologies). qPCR analysis was performed in technical triplicates using biological triplicates. *18 S* rRNA (*Rn18s*) expression was used as a normalisation gene. Data was analysed using the 2^−ΔCt^ method. All primer sequences can be found in Table 1.

### Genomic extraction and polymerase chain reaction (PCR)

~ 80 mg of frozen tissue was homogenised and DNA was extracted as per manufacturer’s instructions in the GenElute™ Mammalian Genomic DNA Minipep Kit. Details of the cycles used can be found in Tables 2 and 3, respectively. Primer sequences used are listed in Table 4. PCR products were electrophoresed on 0.8-2% agarose gels and stained with ethidium bromide staining and visualised using a UV transilluminator or Fujifilm LAS-4000 imager. PCR fragments were purified by gel-extraction for sequence validation (Source Bioscience Ltd).

### Protein extraction and Western blotting

Protein was extracted from ~ 80 mg of tissue in 1 ml of RIPA buffer using sonication. Protein concentration was determined by Bradford assay using a bovine serum albumin (BSA) standard curve. Proteins were separated using sodium-dodecyl-sulphate polyacrylamide gel electrophoresis (SDS-PAGE). Proteins were transferred from SDS-PAGE gels onto nitrocellulose membranes overnight (30 V, 4^o^C) and membranes blocked for one hour using 5% milk at room temperature. After blocking, the membrane was washed three times in 1x phosphate buffered saline (PBS) at room temperature before addition of the primary antibody at a dilution of 1:500. After overnight incubation with rocking at 4^o^C, the membrane was washed three times in excess 1X PBS before addition of the secondary antibody. Antibodies used are listed in Table 5. Specific proteins were detected by chemiluminescence using a Las 4000 imager.

### Statistical analysis

All graphs were generated in Prism, and all statistical analysis was performed in Prism (GraphPad Prism version 8 for Windows, GraphPad Software, La Jolla California USA, www.graphpad.com), with the exception of correlation coefficients and normality tests, which were performed in SPSS (IBM Corp. Released 2019. IBM SPSS Statistics for Windows, Version 26.0. Armonk, NY: IBM Corp). All data is shown as mean ± SEM. Repeated measures three-way ANOVA was performed to analyse circadian and cognitive parameters between genotypes/sex/lighting conditions. Mixed-effects analysis was used when not all subjects completed every repeated measure, e.g. due to FFT-NLLS failings/extreme outliers. Independent T-tests were performed to assess periods/phases for different genotypes/treatments within one lighting condition, total interaction time in one lighting condition, and discrimination indices in one lighting condition. One-sample T-tests were used to compare discrimination indices to 0, to identify if the result was above chance. Paired T-tests analysed sample bias, and object preference. Unpaired T-tests were used to analyse male or female performance across both conditions of light-dark box testing. Bonferroni correction was applied where appropriate.

**Table 1:** qPCR primers.

	qPCR primer	
*Clock* F	CGGCGAGAACTTGGCATT	Exon 15
*Clock* R	AGGAGTTGGGCTGTGATCA	Exon 16
*Bmal1* F	TGACCCTCATGGAAGGTTAGAA	Exon 6 – Exon 7 boundary
*Bmal1* R	GGACATTGCATTGCATGTTGG	Exon 8
*Rev-erbβ* F	GGAGTTCATGCTTGTGAAGGCTGT	Exon 3
*Rev-erbβ* R	CAGACACTTCTTAAAGCGGCACTG	Exon 4
*18 S rRNA* F	GTAACCCGTTGAACCCCATT	Exon 1
*18 S rRNA* R	CAAGCTTATGGCCCGCACTT	Exon 1

**Table 2:** Genotyping PCR cycle details.

95^o^c	30 s	
95^o^c	30 s	X 40 cycles
56^o^c	30 s	
68^o^c	30 s	
68^o^c	5 min	

**Table 3:** cDNA confirmation (*Npas2* exon 3 loss) PCR cycle details.

95^o^c	30 s	
95^o^c	30 s	X 40 cycles
51^o^c	30 s	
68^o^c	30 s	
68^o^c	5 min	

**Table 4:** PCR primers.

	PCR primer
*Cre F*	GCATTACCGGTCGATGCAACGAGTGATGAG
*Cre R*	GAGTGAACGAACCTGGTCGAAATCAGTGCG
*Npas2 lox*P 5’ F	TGGCCAAAGTCTAGCAAAA
*Npas2 lox*P 5’ R	GGAACACACAAGGCACAGGTTA
*Npas2 lox*P 3’ R	GGATGAGTTCCAGGGGATTATGA
*Npas2* exon 2 F	GGACGAAGATGAGAAGGATAGAG
*Npas2* exon 4 R	CTGAGGAATGATGGCTTCCAGT

**Table 5:** Primary antibodies.

	Primary antibody
Npas2	rabbit anti-NPAS2 (HPA019674), Sigma-Aldrich
Actin	mouse anti-β actin (66009-1-IG), Protein Tech

## Electronic supplementary material

Below is the link to the electronic supplementary material.

Supplementary Material 1

## Data Availability

The datasets generated and/or analysed during the current study are available in the University of Nottingham Research data repository, https://rdmc.nottingham.ac.uk. DOI: 10.17639/nott.7455.
