# Supplementary material for: Sex-specific attenuation of constant light-induced memory impairment and Clock gene expression in brain in hepatic Npas2 knockout mice
Source: Sci Rep. 2025 Mar 11;15:8347. doi: 10.1038/s41598-025-92938-1 (PMC11897300; doi:10.1038/s41598-025-92938-1)

Figure S1 **Confirmation of liver specific *Npas2* exon 3 loss in cKO mice.**
A) A schematic detailing the change in DNA band size between wild type *Npas2*, floxed *Npas2* and Cre excision of *Npas2* exon 3. B) Agarose gel of PCR products amplified from the DNA of liver and brain tissue taken from a representative male C57BL/6J mouse, a representative male fl/fl mouse and a representative male cKO mouse. No loss of *Npas2* exon 3 was visible in any DNA extracted from brain tissue, but cKO representative mice demonstrated loss of exon 3 of *Npas2* in the liver.


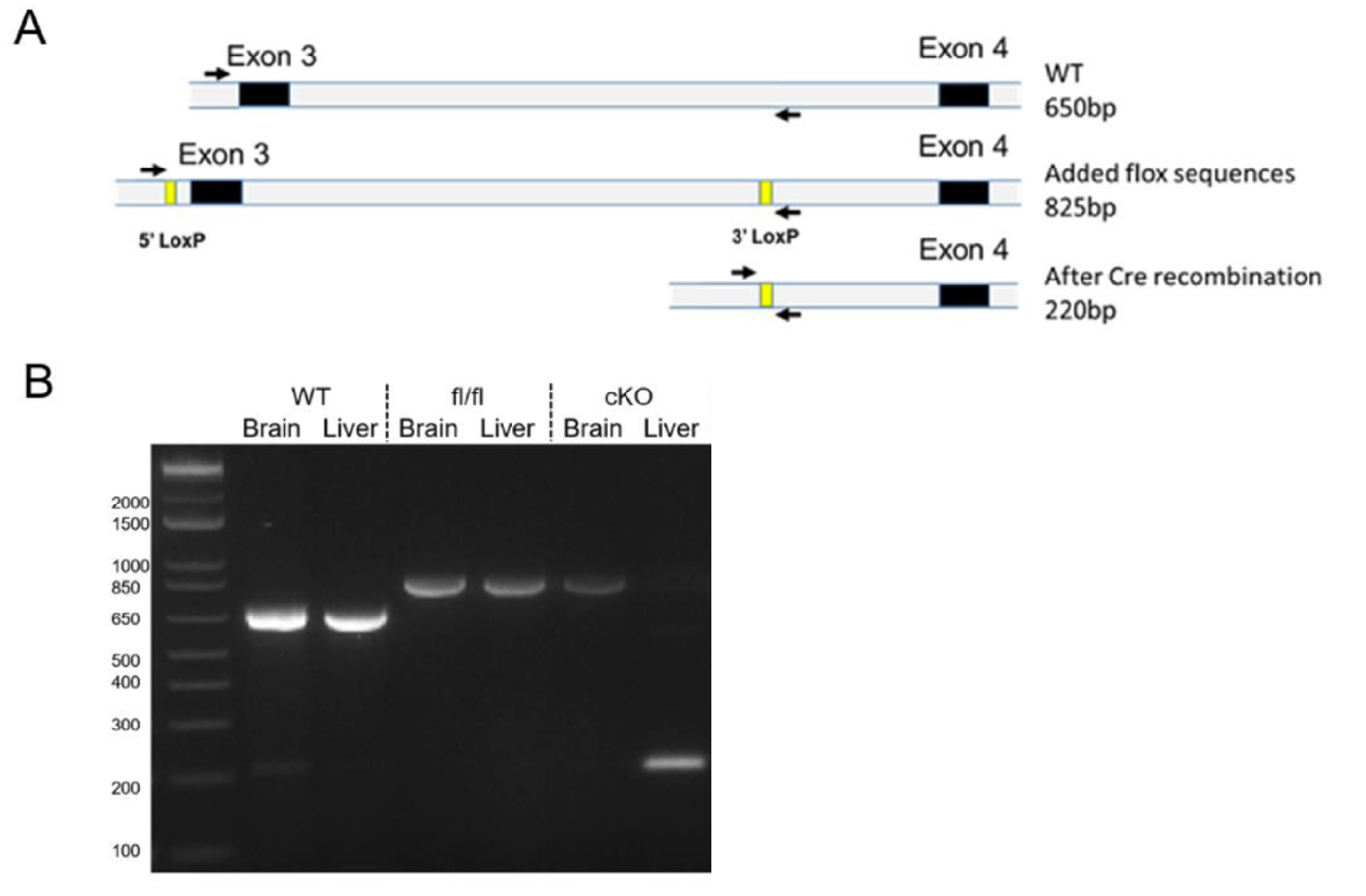


Figure S2 **Significantly reduced NPAS2 levels in cKO mice compared to fl/fl controls.** A) Western blots detecting NPAS2 revealed a reduction in protein level in the liver of female cKO mice compared to the liver of female fl/fl mice. Expected NPAS2 band size is 92 kDa. Each sample represents the liver of one mouse. C) Five repeated densitometry measurements showed significantly higher NPAS2 levels in fl/fl females compared to cKO females (****, *p*<0.0001).


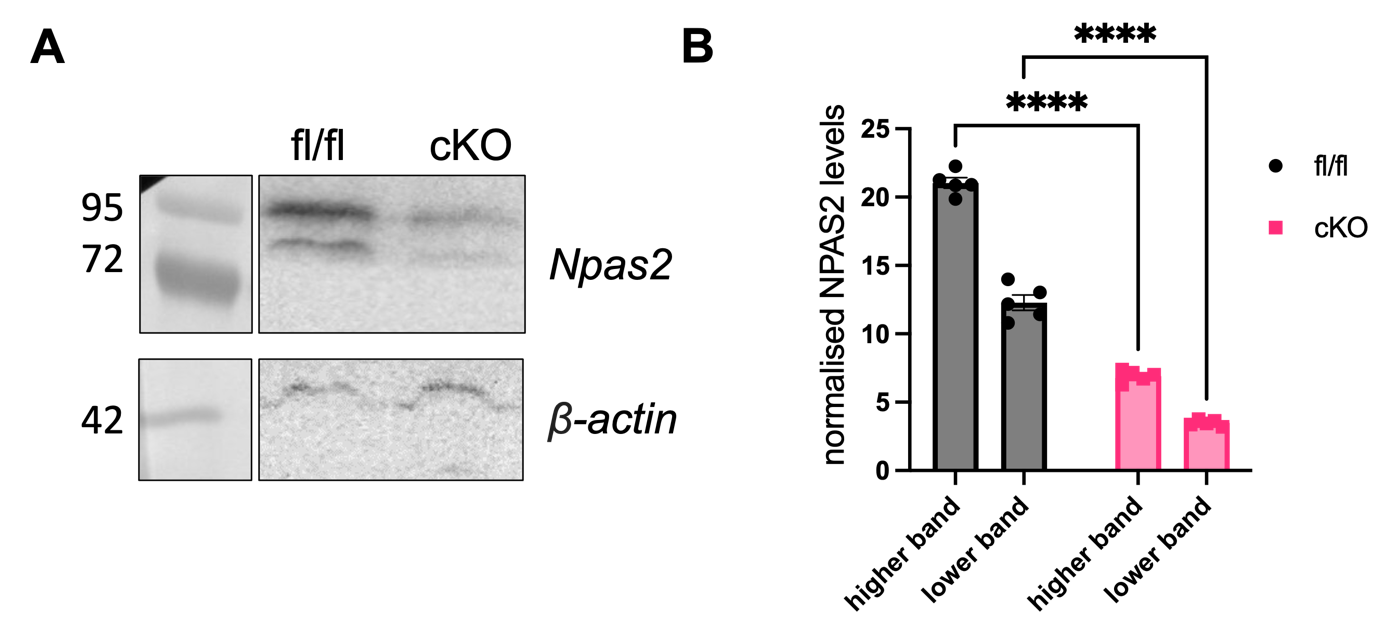


Figure S3 **No location preference shown in either genotype during sample trial.** A) Fl/fl controls (LD n = 15, DD n = 17, LL n = 17) showed no significant preference in object location in sample trials under any lighting condition. B) cKO mice (n = 14 in LD, n = 15 in DD and LL) showed no significant preference in object location in sample trials under any lighting condition


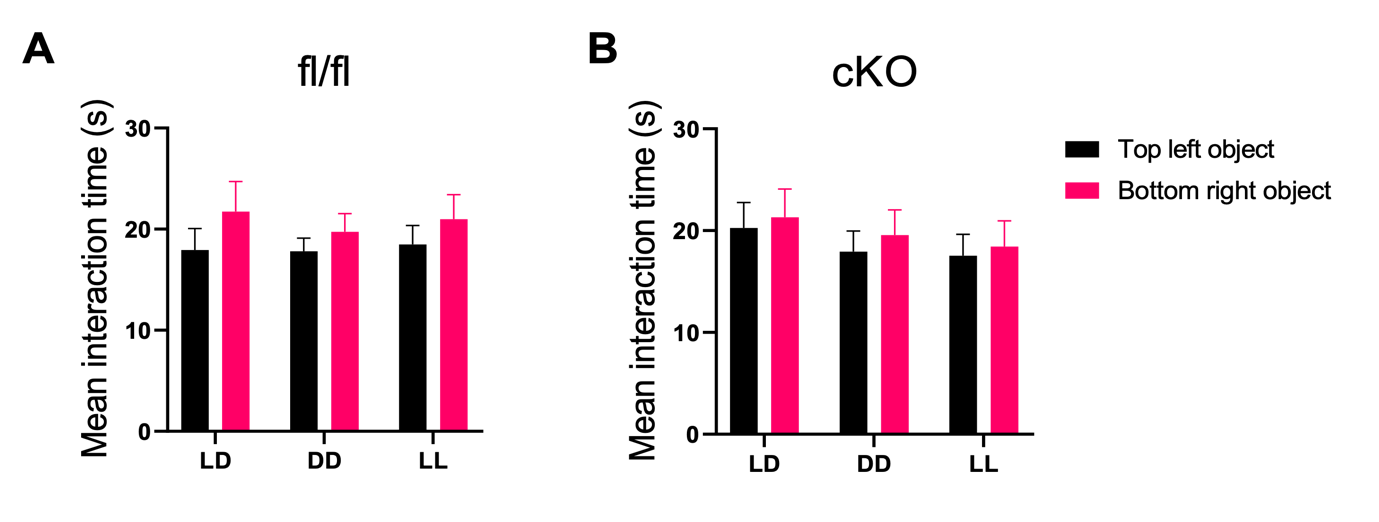


Figure S4 Actograms of locomotor activity across three light conditions (Fl/Fl males)


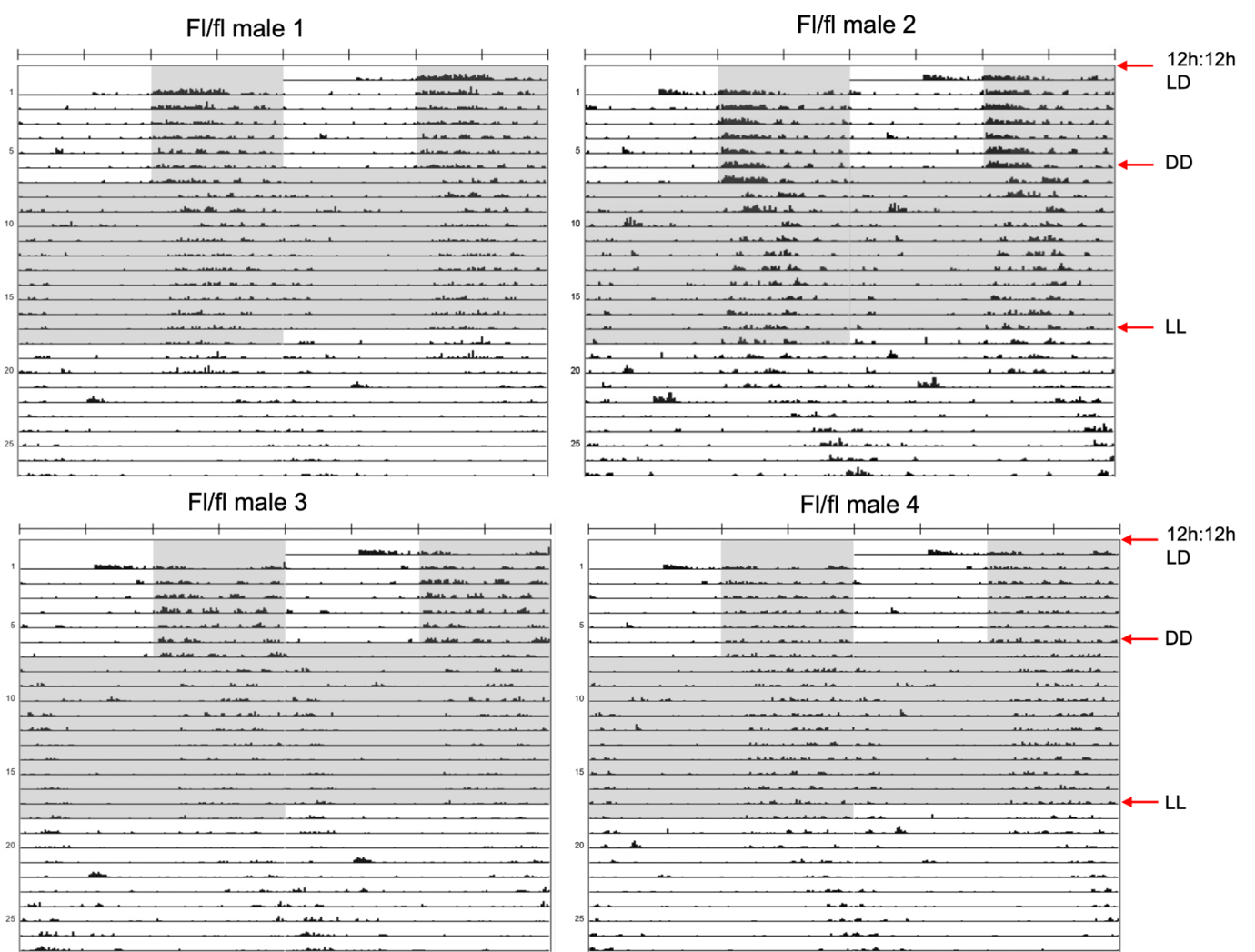


Figure S5 Actograms of locomotor activity across three light conditions (Fl/Fl females)


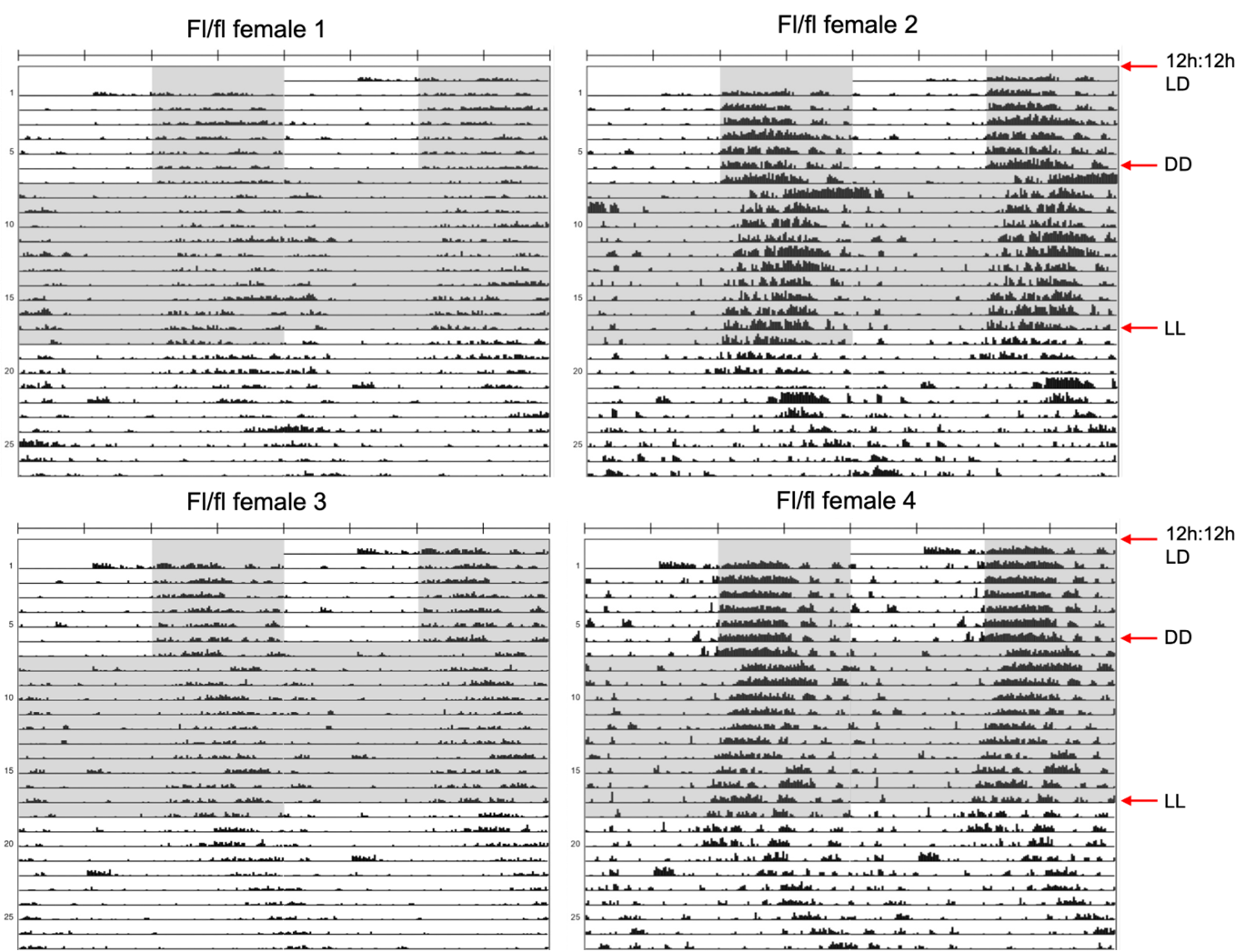


Figure S6 Actograms of locomotor activity across three light conditions (cKO males)


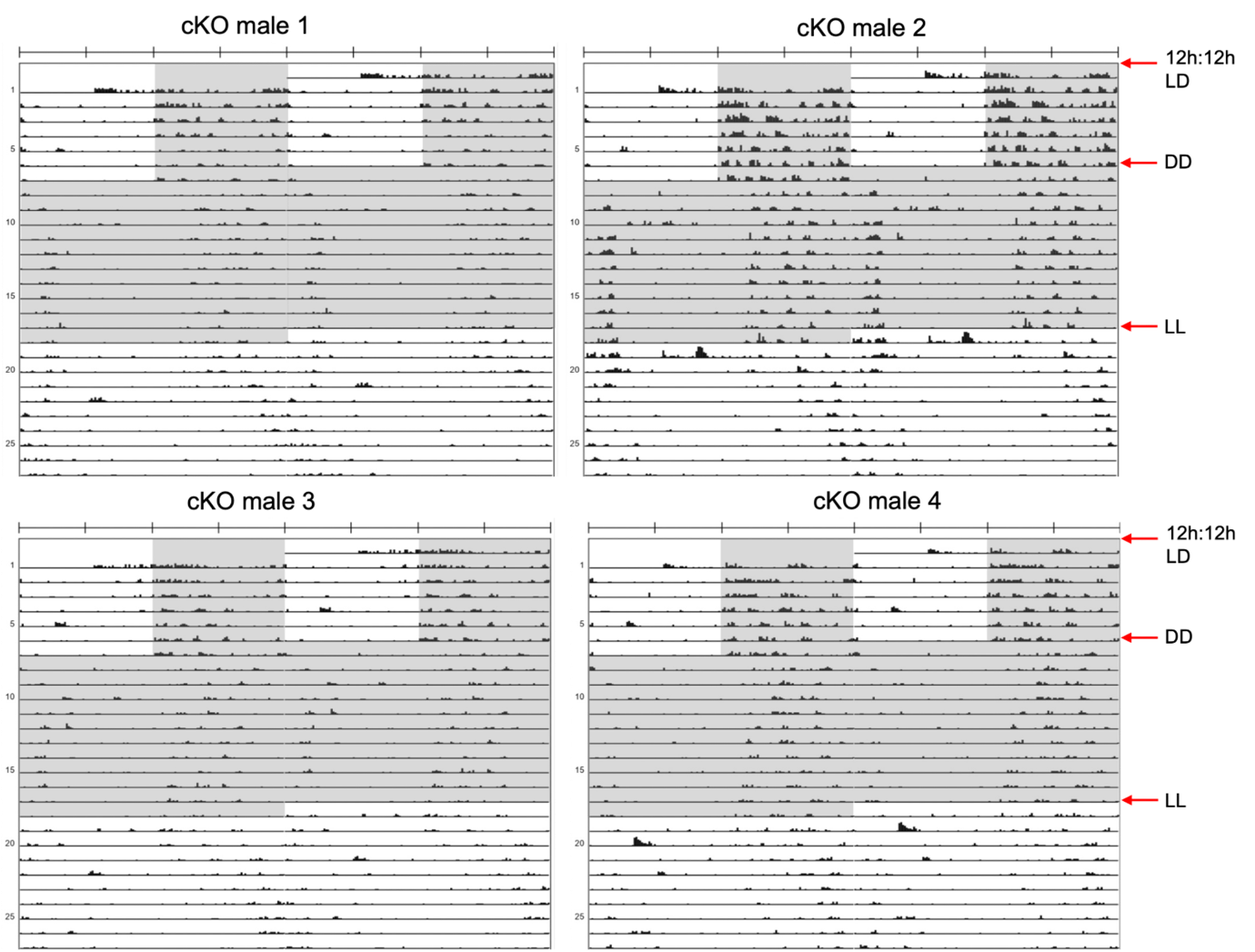


Figure S7 Actograms of locomotor activity across three light conditions (cKO females)


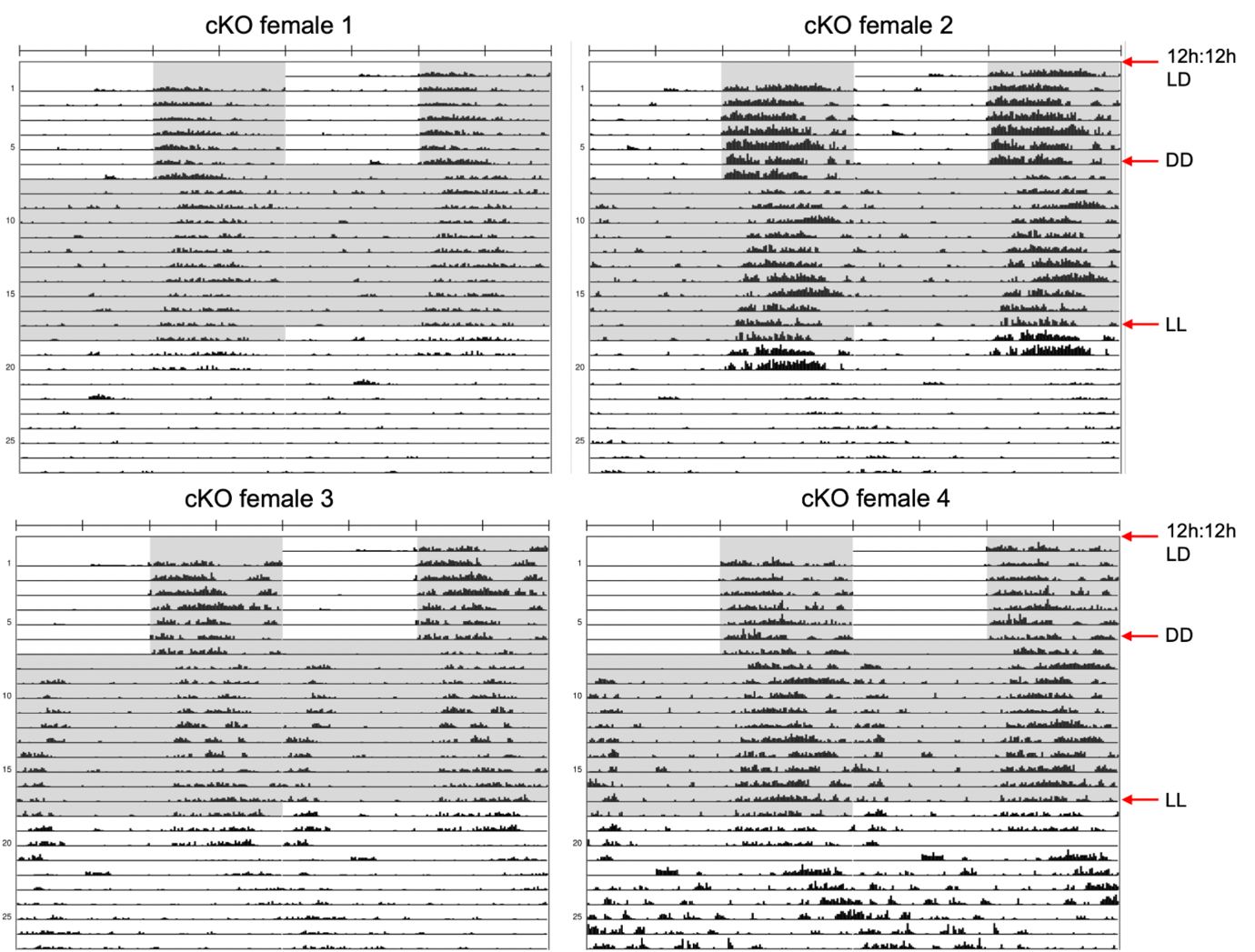


Figure S8 **Both genotypes display equal levels of anxiety in all parameters of the open field arena trial.** A) Both genotypes (fl/fl n = 12, cKO n = 14) had a significant preference for the peripheral zone of the open field arena, over the central zone (both *****p*<0.0001). B) No significant differences were seen between genotypes in the time spent in the central zone. C) No significant differences were seen between genotypes in the total distance travelled over the test period.


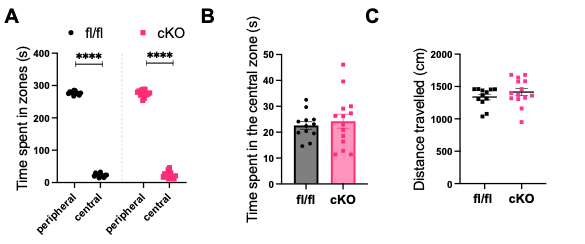


Figure S9 **Both genotypes display equal levels of anxiety in all parameters of the light-dark box trial.** A) No significant differences between genotypes (fl/fl n = 12, cKO n = 14) were noted in latency to enter the dark chamber of the light-dark box. B) No significant differences in number of transitions across chambers between genotypes (fl/fl n = 12, cKO n = 13). C) No significant differences between time spent in the dark chamber was seen between genotypes (fl/fl n = 15, cKO n = 16). D) No significant differences in number of aborted entries into the dark chamber between genotypes (fl/fl n = 12, cKO n = 13).


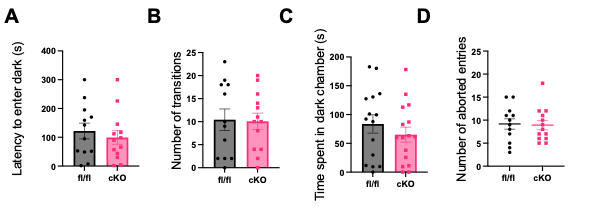


Figure S10


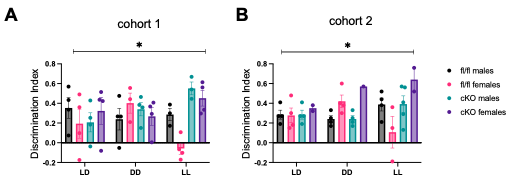


Figure S11 No sex differences in discrimination index in Wild-Type C57BL/6 mice in LD conditions but a trend towards male superiority on DI as has been reported in c57/BL6 mice (Frick & Grisack 2003, Behavioral Neuroscience Vol. 117, No. 6, 1283–1291 but not in other studies (Bettis and Jacobs (2009) Behavioural Processes 82 249-255). No significant differences between male (n = 8) and female (n = 5) DIs were found during short-term NOR tests (fig. A). WT females show a significantly higher mean total interaction time during the NOR test (***p*=0.0080) (fig. B).


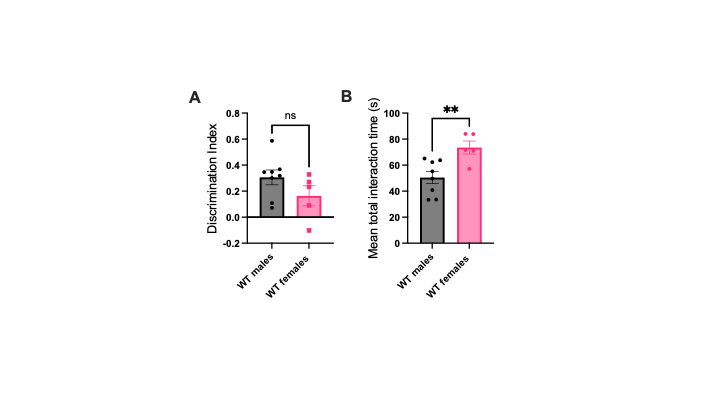

Supplement: Supplementary file 1 — Supplementary Material 1 [file 41598_2025_92938_MOESM1_ESM.docx]
